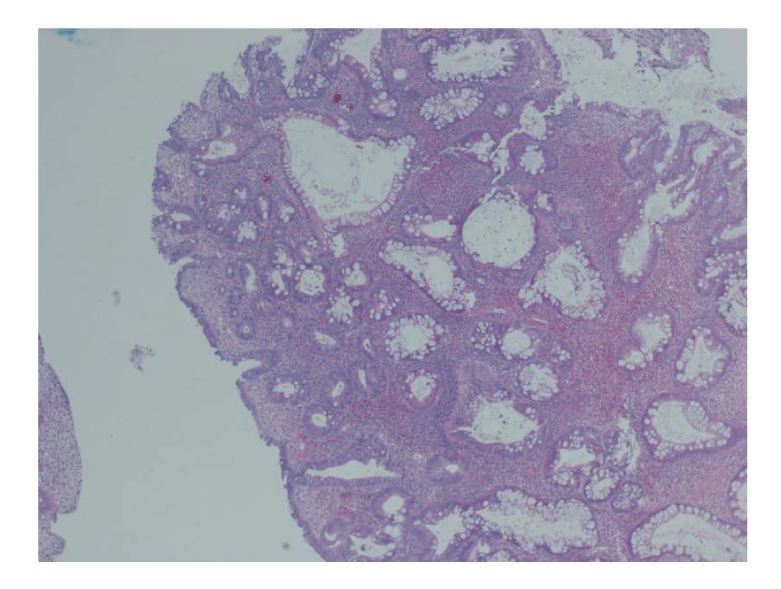# Cronkhite-Canada Syndrome: A Case Report and Review of Literature

**DOI:** 10.1155/2009/619378

**Published:** 2009-08-25

**Authors:** Kevin T. Kao, Jitesh K. Patel, Vijayamalini Pampati

**Affiliations:** ^1^Department of Gastroenterology, Kaiser Permanente Los Angeles Medical Center, 1526 N. Edgemont Street, Los Angeles, CA 90027, USA; ^2^Department of Gastroenterology, Kaiser Permanente Woodland Hills Medical Center, 5601 De Soto Ave, Woodland Hills, CA 91367, USA

## Abstract

Cronkhite-Canada syndrome (CCS) is a rare syndrome first described in 1955. (1) Since then, 400 cases worldwide have been reported in the literature. The disease is characterized by diffuse gastrointestinal polyposis, dystrophic changes of the fingernails, alopecia, cutaneous hyperpigmentation, diarrhea, weight loss, and abdominal pain. (2) The etiology is currently unknown, but an autoimmune process is suspected. The workup is based on history and physical followed by imaging and endoscopy with biopsy to confirm gastrointestinal polyposis. The goal of treatment focuses on symptomatic management of the patient and nutritional support.

## 1. Introduction

Cronkhite-Canada syndrome (CCS) is a rare disease characterized by the presence of diffuse gastrointestinal polyposis, dystrophic changes in the fingernails, alopecia, cutaneous hyperpigmentation, diarrhea, weight loss, and abdominal pain. Since 1955 when the syndrome was first documented, only about 400 cases have been reported worldwide. Here we report a case of CCS in a woman of Filipino decent.

## 2. Case Presentation

### 2.1. History of Present Illness

M. N. is a 39-year-old Filipino woman with a prior history of intestinal amebiasis, who was referred to gastroenterology for evaluation of epigastric pain. Her symptoms began two months prior to presentation with 2 weeks of watery diarrhea for which she was treated with metronidazole for presumed amebiasis. She reported improvement of her diarrhea but subsequently developed postprandial epigastric pain described as sharp in nature and lasting for hours. She denied any fever, chills, nausea, vomiting, dysphagia, or odynophagia. However, she did report decreasing appetite with early satiety and a 9 kg weight loss over one month. She was initially evaluated by her primary care physician, who prescribed famotidine for her epigastric pain as well as hydrochlorothiazide for new onset hypertension. Shortly after this visit, she noticed a significant amount of hair loss as well as hyperpigmentation of her palms and soles. She worked at a jewelry store and denied any substance abuse. Her family history was significant for one uncle who had colon cancer at an unknown age.

### 2.2. Physical Exam

**Figure 1:**
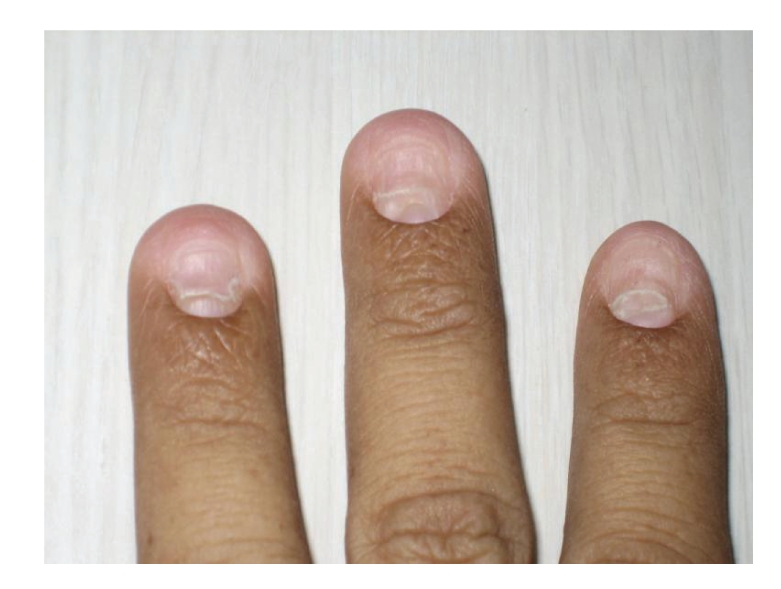


**Figure 2:**
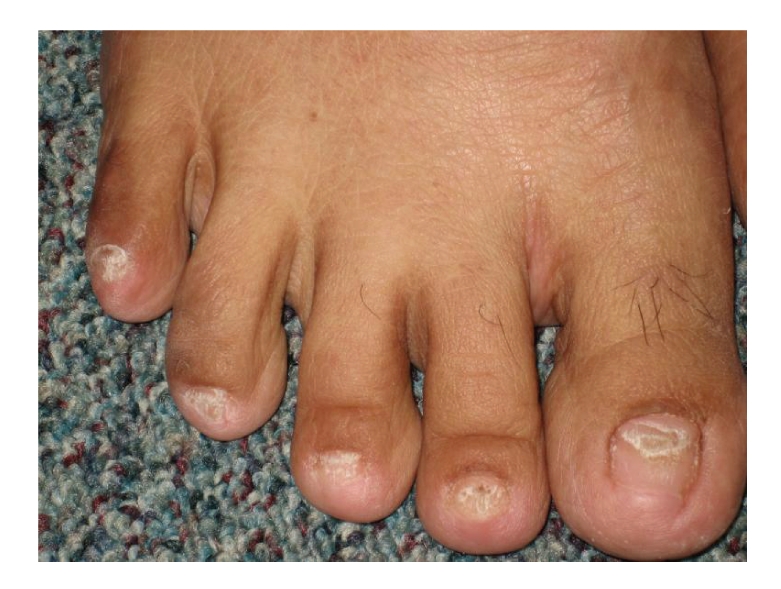


**Figure 3:**
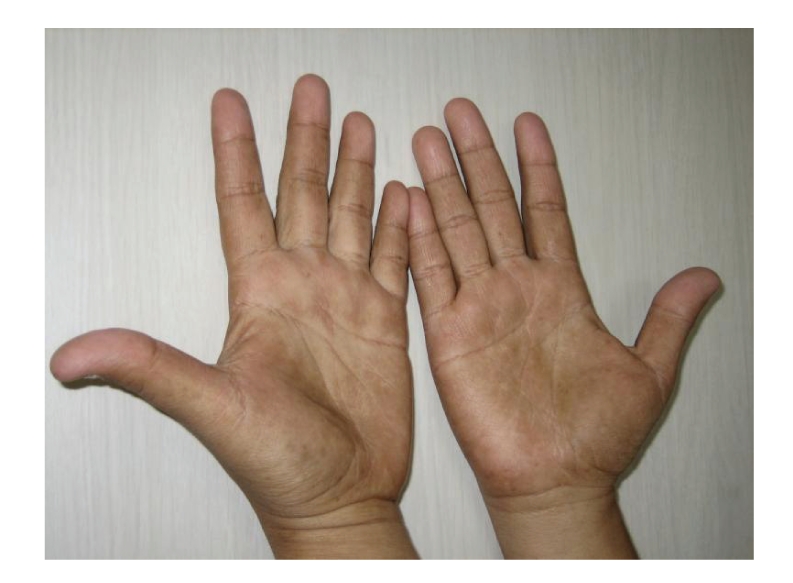


Patient's vital signs were unremarkable. Her weight was 68 kg and her height was 1.6 meters. Her exam was significant for diffuse alopecia and dystrophic changes of her fingernails (see Figure 1) and toenails (see Figure 2). There was also a faint hyperpigmentation of her palms (see Figure 3) and soles. She had no lymphadenopathy and her abdominal exam was unremarkable.

### 2.3. Laboratory

Her white blood cell count was 12.3 thousand cells/mL (normal range 4–11 thousand cells/mL) with an absolute eosinophil count of 233 cells/mL. Her hemoglobin was 15.2 gm/dL (normal range 12–16 gm/dL) and hematocrit was 44.5% (normal range 37–47%). Albumin was 3.2 gm/dL (normal range 3.3–4.8 gm/dL) and her aminotransferases were normal.

### 2.4. Endoscopy Result

**Figure 4:**
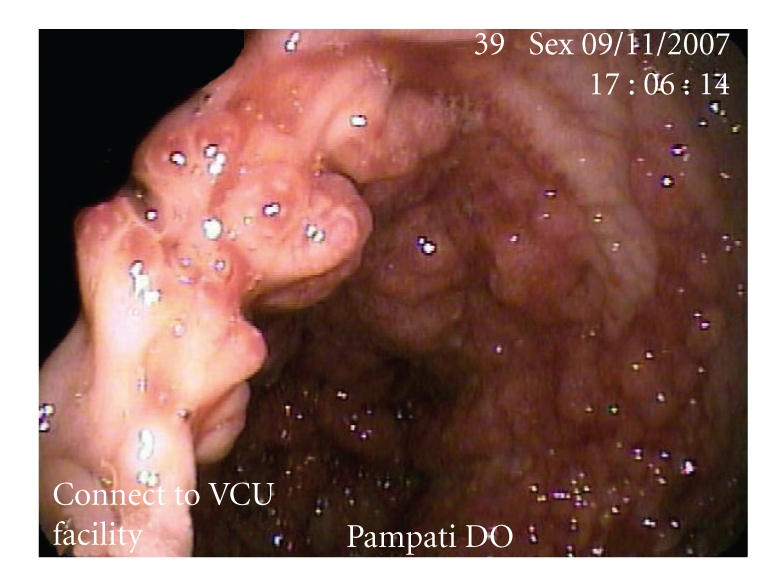


**Figure 5:**
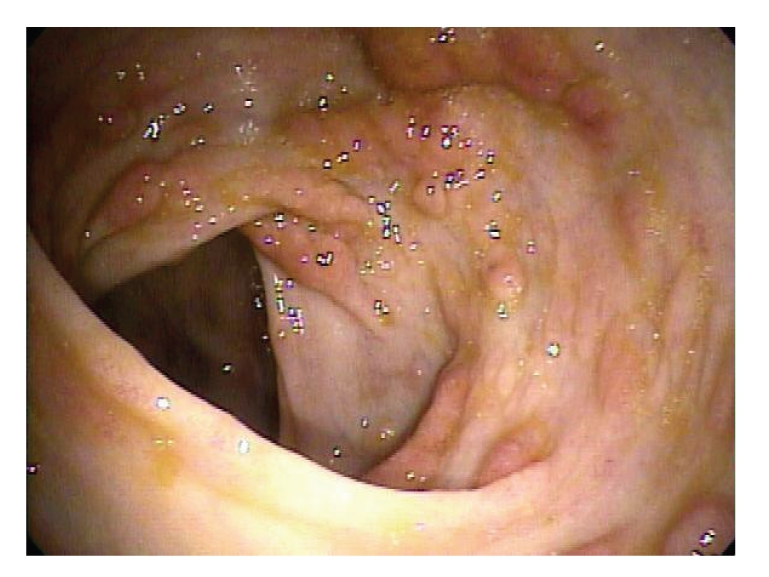


Esophagogastroduodenoscopy revealed diffuse sessile polypoid lesions within the stomach. They ranged between 4 mm to 10 mm in size with mucosal edema. There did not appear to be a clear cut separation of the polyps from the surrounding mucosa (see Figure 4). In contrast to EGD, colonoscopy revealed the colonic mucosa carpeted with sessile, strawberry-like polypoid lesions, 2 mm to 10 mm in size, but with normal appearing surrounding colonic mucosa (see Figure 5). Multiple polyps within the stomach and colon were removed along with normal appearing mucosa and sent to pathology.

### 2.5. Pathology

The colonic polyps revealed adenomatous changes with stromal edema and dilated glands. The gastric polyp biopsies showed focal hyperplastic features with dilated glands. There was no evidence of eosinophilia (see Figure 6).

### 2.6. Treatment Course

CCS was diagnosed based on a combination of her symptoms, clinical features, and the histopathology of polypoid lesions seen during endoscopy. Additional blood work obtained including immunoglobulin gamma 4 (IgG4) and Antineutrophilic cytoplasmic antibodies (ANCAs) were all within normal limits. Due to her weight loss and poor appetite, nutritional therapy was initiated in the form of total peripheral nutrition (TPN). At one month followup, the patient reported a weight gain of 6 kg. She also reported improvement of her alopecia, nail dystrophy, and symptoms of diarrhea. TPN therapy was discontinued after 2 months when she reported a rash around the infusion site. Steroid therapy was discussed with the patient but was declined. At her last followup, 12 months after the initial diagnosis, she appeared to be in clinical remission. Repeat endoscopy after 12 months also showed significant improvement in the number of polyps seen.

## 3. Discussion

Cronkhite-Canada syndrome is a rare systemic disease first reported in 1955 by Cronkhite and Canada [1]. Since then, around 400 cases have been reported worldwide with the Japanese contributing over 75% of these case reports [2]. Patients of European or Asian descent are most frequently affected. The estimated incidence of CCS is one per million based on the largest study performed to date [3]. The mean age of onset is estimated to be in the fifth to sixth decade, with a slight male predominance in 3 : 2 ratio [4].

The etiology of CCS is currently unknown. So far, there is no strong evidence to suggest a familial predisposition. Interestingly, cases have been associated with elevated antinuclear antibody (ANA) and IgG4 levels [2, 5, 6]. There is also an association between CCS to hypothyroidism [7] and various autoimmune diseases such as systemic lupus erythematous, rheumatoid arthritis, and scleroderma, all of which point toward an autoimmune etiology [8]. However, others have reported mental stress and physical fatigue may also contribute to the etiology [3].

The symptoms of CCS can vary, but classically it is characterized by the presence of diffuse gastrointestinal polyposis, dystrophic changes in nails, alopecia, cutaneous hyperpigmentation, diarrhea, and weight loss. Other symptoms such as hypogeusia and xerostomia have also been described in literature [9]. Endoscopic appearance of CCS varies according to current literature. Gastric mucosa has been described as being thickened hypertrophic gastric folds mimicking Menetrier's disease to atrophic appearing with polypoid lesions [10]. Colonic polyps have been characterized as sessile and “strawberry like” in one study [11]. 

The optimal treatment of CCS is currently unknown due in part to its rarity. Nutritional support aimed at improving electrolytes, vitamin, and mineral deficiencies can rarely induce complete remission as seen in our patient. In fact, current literature favors a combination therapy based on nutritional support and corticosteroids [12]. We believe that TPN therapy in combination with partial bowel rest may have provided crucial nutritional support allowing the disease process to enter remission. Other therapies such as antihistamine receptor antagonist agents and cromolyn sodium have also been used as supplement therapy in cases where degranulating eosinophils and mast cells are seen on biopsy [13]. One Case report has also suggested improvement of CCS after eradication of Helicobacter Pylori infection [14]. The total treatment period is not well defined, varying from 6 to 12 months of combination therapy.

Numerous complications can rise from CCS, with the most notable being the development of malignancy. This can be as high as 15% [15]. Both gastric and colorectal cancers have been reported, with sigmoid colon and the rectum being the most common initial site of cancer [16]. Unfortunately, due to the rarity of this disease, optimal screening protocols have not been developed, although annual endoscopic surveillance has been widely practiced. 

The long-term prognosis is quite poor according to early studies. One study is reporting a 55% mortality rate in their 55 patient case study [8]. However, with improvement in medical therapy and increased recognition of the syndrome, the prognosis is now thought to be better compared to earlier case reports.

## Figures and Tables

**Figure 6:**